# Feasability of a Frameless Brain Biopsy System for Companion Animals Using Cone-Beam CT-Based Automated Registration

**DOI:** 10.3389/fvets.2021.779845

**Published:** 2022-02-09

**Authors:** Felix Meneses, Arianna Maiolini, Franck Forterre, Anna Oevermann, Daniela Schweizer-Gorgas

**Affiliations:** ^1^Division of Clinical Radiology, Department of Clinical Veterinary Medicine, Vetsuisse-Faculty, University of Bern, Bern, Switzerland; ^2^Division of Neurology, Department of Clinical Veterinary Medicine, Vetsuisse-Faculty, University of Bern, Bern, Switzerland; ^3^Division of Small Animal Surgery, Department of Clinical Veterinary Medicine, Vetsuisse-Faculty, University of Bern, Bern, Switzerland; ^4^Neurocenter, Department of Clinical Research and Veterinary Public Health, Vetsuisse-Faculty, University of Bern, Bern, Switzerland

**Keywords:** CBCT-based automated registration, brain biopsy, frameless, dogs, cats

## Abstract

The aim of the present study was to evaluate the use of a novel intraoperative cone-beam computed tomography (CBCT)-based automated registration system for frameless stereotactic brain biopsy in companion animals. An experimental cadaveric study evaluated thalamic and piriform lobe target site needle placement error in three dogs and three cats without a history of intracranial disease. Diagnostic accuracy and diagnostic yield were prospectively evaluated in twenty-four client-owned dogs and four cats with intracranial disease. Twenty-one procedures were performed *post mortem* (eighteen dogs and three cats), and seven biopsy procedures were performed in alive patients (six dogs and one cat). Procedural duration was evaluated in ten post mortem and four living patients. Outcome was evaluated in six dogs and one cat. In dogs, the calculated median needle placement error was 1.8 mm (range 0.71–2.84 mm) and 1.53 mm (range 1.45–1.99 mm) for piriform lobe and thalamus target sites, respectively. In cats, the calculated median needle placement error was 0.79 mm (range 0.6–1.91 mm) for the piriform lobe target site and 1.29 mm (range 0.47–2.69 mm) for the thalamic target site. The diagnostic yield was 96.4% (95% CI 0.81–0.99), the diagnostic accuracy was 94.4% (95% CI 0.72–0.99). Median total procedural duration for post mortem biopsies was 57.5 min (range 41–69 min). Median total procedural duration for *intra vitam* biopsies was 122.5 min (range 103–136 min). Three dogs were discharged 1 day after biopsy and one dog after 6 days. Two dogs and one cat were euthanized 24 and 48 h after biopsy. Intraoperative CBCT-based automated image registration for frameless stereotactic biopsies in companion animals is capable of providing diagnostic brain biopsy specimens independent of skull size and morphology with diagnostic yield and accuracy comparable to published values for diverse frameless and frame-based stereotaxy systems used in veterinary medicine. Duration of the procedure is not negatively affected and within the published range with other systems. Mobile intraoperative CBCT-based registration combined with neuronavigation delivers diagnostic brain biopsies in companion animals.

## Introduction

Recent advances in imaging technology and therapeutic modalities have led to significant improvements in the treatment of intracranial disease in companion animals (1). However, a histological diagnosis before treatment remains the exception, and the majority of evidence-based studies favoring one treatment modality over another are based on a presumptive diagnosis (2).The ability of magnetic resonance imaging (MRI) to differentiate vascular, inflammatory, and neoplastic disease is deceptively low (3–5). In people, the discordance of histological and imaging diagnosis based on MR imaging varies from 16 to 30% (6, 7) leading to treatment changes in 16–27% (8). Therefore, an aggressive treatment with concomitant side effects should be based on brain biopsies (9). A wide variety of stereotactic brain biopsy systems are currently successfully employed in veterinary clinical practice (10–21). Brain biopsy systems with stereotactic guidance are either frame-based or frameless, with the former considered to be the standard of care due to its excellent targeting precision. Frame-based stereotaxy uses a rigid skull-mounted frame that functions as a coordinate system to which any point inside the brain can be referenced. Frameless stereotaxy dispenses of the bulky frame and uses anatomical landmarks or skin fiducials to register image coordinates to patient coordinates instead. The advantages of frameless stereotaxy include minimally invasive technique and reduced post-operative infection risk, improved target visualization and accessibility, flexibility to sample multiple targets, and reduced procedure time (22). In conjunction with adjustable rigid aiming devices, target accuracy and diagnostic yield are nowadays equivalent to frame-based stereotaxic systems (22, 23).

In recent times, compact mobile computed tomography (CT) imaging units combined with navigation systems are increasingly used for precision neurosurgical and orthopedic interventions (22, 24). Newer units are equipped with an integrated indicator box that allow intraoperative image acquisition with immediate and automatic registration of the patient to the images (25, 26). In veterinary medicine utilization of a mobile cone-beam CT (CBCT) system has been described for computer-assisted surgery in horses (24). Adapting this system for brain biopsies in dogs and cats has the potential to simplify brain biopsy procedures, decreasing procedure times and registration inaccuracies by eliminating the need for patient transport between the imaging and operating rooms as well as user-dependent patient registration. Data regarding needle placement error, diagnostic yield, diagnostic accuracy, procedural time, and outcome with this system in this setting are currently lacking. The aim of the present study was to report our experience adapting an intraoperative CBCT-based automated registration system for frameless brain biopsies in companion animals. We hypothesized that needle placement error, diagnostic yield, and diagnostic accuracy would be comparable to previously reported brain biopsy systems and that procedural times would be shorter.

## Materials and Methods

The first part of the study consisted of a prospective experimental cadaver study in dogs and cats to test the needle placement error. The second part consisted of a prospective study in dogs and cats where an intracranial lesion was diagnosed by MRI or CT and a brain biopsy was conducted, either in alive patients or *post mortem*. All interventions were performed with signed owner consent.

### Experimental Cadaver Study

**Figure 1:**
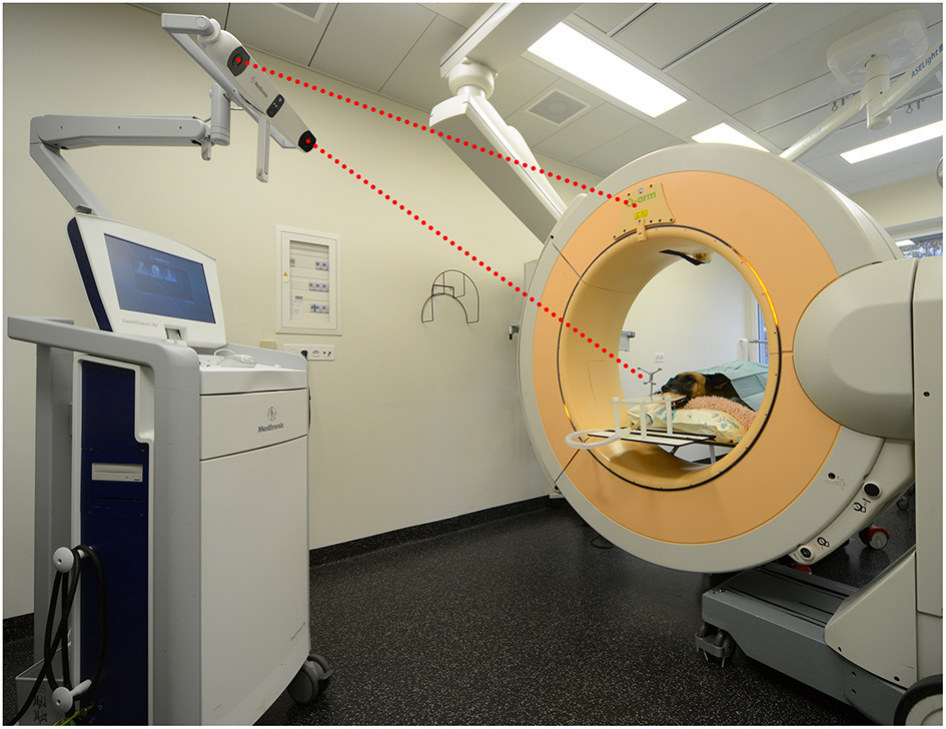
Set up of the mobile cone-beam computed tomography and navigation system in the operating room showing a dog cadaver in sternal recumbency. The head has been secured to the bite plate. For image registration to the patient's anatomy, a head scan is performed during which the infrared camera must see the reference array and indicator box of the scanner (red dotted lines).

Needle placement error was tested in three dogs and three cats without a history of intracranial disease that were euthanized for reasons unrelated to the study. A 3D T1-weighted gradient echo sequence (TE 6.91 ms, TR 25 ms, FA 30°, matrix between 320 × 320 and 576 × 576, FOV between 56.67 and 66 mm, slice thickness 1 mm) was acquired using a 1.0T unit within 24 h of euthanasia. The cadavers were transported to the operating room and positioned in sternal recumbency on a carbon table. Memory foam pads were used to stabilize the trunk and neck. Heads were clipped and the maxillary dental arcades firmly secured with dental putty (President, Coltene, Altstatten, Switzerland) to a custom-made perforated bite plate with an attached reference array equipped with infrared reflectors (Medtronic Schweiz AG, Münchenbuchsee, Switzerland). A single high resolution 26 second head scan was acquired with a volume of 22 × 22 × 17 cm 3 and a pixel size of 0.415 mm 2 (O-arm, Medtronic Schweiz AG, Münchenbuchsee, Switzerland) with both reference array and indicator box of the scanner in the field of view of the infrared navigation camera of the dedicated workstation (StealthStation S7, Medtronic Schweiz AG, Münchenbuchsee, Switzerland) (Figure 1). The volumetric acquisition was then transferred to the workstation for automated patient registration and automated image fusion (StealthViz, Medtronic Schweiz AG, Münchenbuchsee, Switzerland) with the preloaded MRI 3D T1-weighted MRI series. Correct alignment of fused images was evaluated visually on the workstation. Target points and trajectories were planned on the merged dataset (Framelink 5.0, Medtronic Schweiz AG, Münchenbuchsee, Switzerland). In each cadaver, one target site was set in the left piriform lobe and one in the right thalamus. Entry sites were planned to be perpendicular to the skull, with resultant trajectories avoiding sulci and the ventricular system. Throughout the procedure the navigation camera was positioned such that the reflective spheres of the reference array and navigable instruments were visible at all times. Registration accuracy was controlled regularly on anatomical landmarks. An articulating instrument holder (Vertek single-lever articulating support arm, Medtronic Schweiz AG, Münchenbuchsee, Switzerland) attached to the surgical table and a navigable pointer (Vertek, Medtronic Schweiz AG, Münchenbuchsee, Switzerland) were then adjusted with real-time visual feedback to reproduce the planned trajectory in the navigable space. Once the position corresponded to the planned trajectory, the articulating arm was locked in position. A standard surgical rostrotentorial approach was used to expose the skull. A high-speed pneumatic drill with a 3 mm burr head was used to create a burr-hole and the dura mater was incised with a dedicated dura perforator or a 18 G hypodermic needle with bent tip. The navigable pointer was then exchanged for a navigable, 2 × 200 mm, Sedan-type brain biopsy needle (model 9733068, Medtronic Schweiz AG, Münchenbuchsee, Switzerland). This biopsy needle is equipped with two spherical infrared reflectors that allow direct feedback of needle tip and biopsy window positions by the navigation system. The biopsy needle was then advanced under real-time visual feedback toward the planned target site, and a second volume acquisition with the brain biopsy needle in place was performed. After merging the images, the mean needle tip deviation was measured in transverse, dorsal, and sagittal planes from the superimposition of the planned trajectories with the actual needle position on the workstation. The Euclidean distances were then determined using the formula:


$$\begin{array}{l} Error\;=\;\surd \left[{\left(\Delta x\right)}^{2}\;+\;{\left(\Delta y\right)}^{2}\;+\;{\left(\Delta z\right)}^{2}\right] \end{array}$$

### Clinical Patients

**Figure 2:**
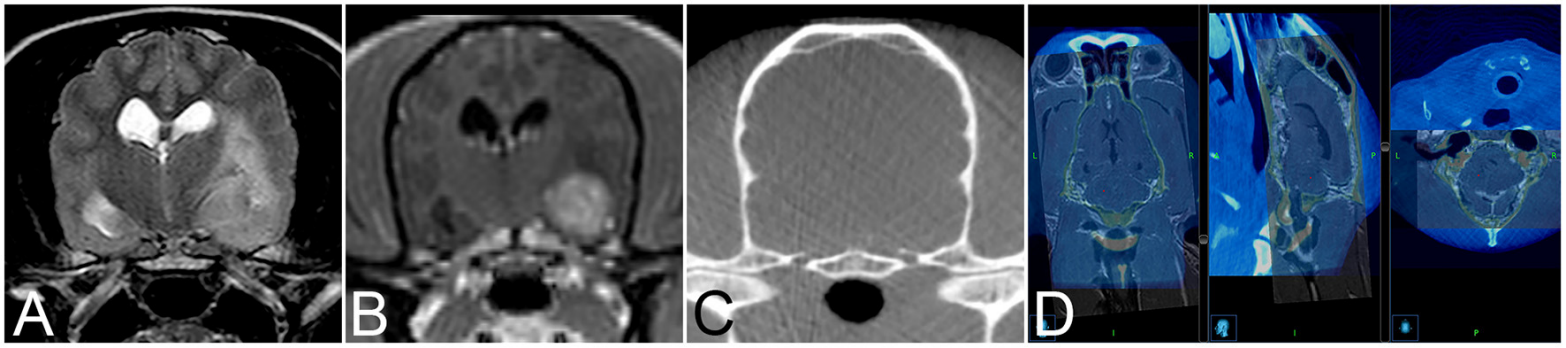
Transverse T2-weighted **(A)** and T1-weighted contrast enhanced **(B)** image and of an 11-year-old female spayed Poodle with biopsy proven vasculitis and meningoencephalitis of unknown origin. Corresponding intraoperative cone beam computed tomography (CBCT) image **(C)** for image fusion. **(D)** Example of image fusion of the CBCT images with magnetic resonance images of a 9-year-old female spayed German Shepherd Dog with a biopsy proven WHO grade II cerebellar diffuse astrocytoma.

**Figure 3:**
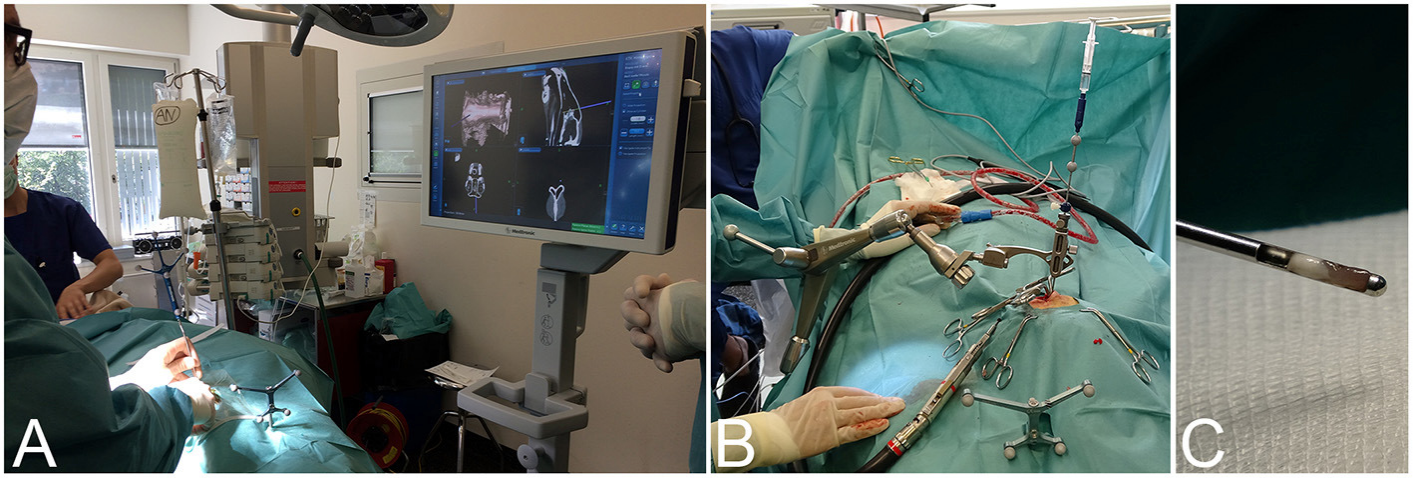
Intraoperative workflow for navigated frameless stereotactic brain biopsy. **(A)** Finding the optimal skin incision site to match the entry point using the navigable pointer with real-time visual feedback on the system's surgeon monitor. **(B)** After dura opening, the navigable biopsy needle has been advanced to the target depth under visual guidance. **(C)** Example of a brain biopsy specimen taken from the periphery of a lesion.

Twenty-four client-owned dogs and four cats presented to the neurology service of the Vetsuisse-Faculty Bern during the period between July 2017 and July 2020 that underwent brain biopsy as part of the diagnostic work-up of clinical signs referable to intracranial disease were prospectively included in this study. Signalment, presenting complaint, neuroanatomical localization based on neurological exam, and outcome were recorded. General anesthesia for dogs and cats undergoing *intra vitam* biopsy was performed by the anesthesiology service and protocols varied based on their individual assessment of each patient. The heads of animals were clipped, aseptically prepared, covered, and patients subsequently transported to the operating room. The animals were then positioned and immobilized as described for the cadaveric study. Following the O-arm acquisition a second surgical skin preparation was done, and sterile draping started while automated patient registration, automated image fusion with a preloaded preoperative contrast enhanced CT or 3D T1-weighted MRI series, and trajectory planning were done on the workstation in the operating room (Figure 2). Biopsy trajectory planning was performed on the merged dataset defining both target and entry sites depending on lesions location and MRI features (Figure 3A). As previously described, trajectories were planned perpendicular to the skull avoiding sulci and ventricular system, except in animals with intraventricular lesions. Target sites were selected to include contrast enhancing areas of the lesions when present, avoiding cystic, necrotic, or hemorrhagic areas. The articulating instrument holder was attached to the surgical table on the same side of the brain lesion. Instrument holder and navigable pointer were then adjusted with real-time visual feedback and standard surgical rostro- or caudotentorial approaches and dura mater incision were performed as described before. The pre-calibrated brain biopsy needle was then advanced under real-time visual feedback until the biopsy window was centered on the planned target (Figure 3B). Two to six biopsies were taken at different depths and orientations, with the number of biopsies depending on the tissue yield (Figure 3C). The bone defect was left uncovered, muscles and skin were adapted following standard closing procedures. Dogs and cats undergoing post mortem biopsy were prepared and positioned as for the cadaveric study and biopsies were taken as for the intra vitam patients.

### Procedural Duration Measurements

**Figure 4:**
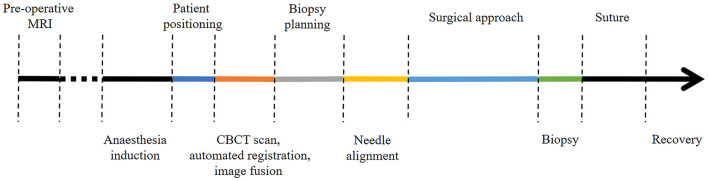
Color coded timeline showing the different procedural steps. The time intervals of the colored procedural steps were recorded.

Minor adjustments involving optimization of the spatial disposition of the CBCT and workstation in the operating room and of the image-guided articulating instrument holder on the surgical table were performed during the first three intra vitam procedures. Thereafter, to investigate procedural duration five time intervals were determined and recorded for patient positioning, including CBCT image acquisition in the operating room, trajectory planning, biopsy needle alignment, surgical approach, and biopsy retrieval in four intra vitam and ten post mortem biopsy procedures (Figure 4). The total procedural duration defined as start of patient positioning to end of biopsy specimen retrieval resulted from adding all intervals.

### Histopathological Examination

Biopsy specimens were flushed into biopsy cassettes or nylon mesh biopsy bags. Biopsies and brains were fixed by immersion in 10% buffered formalin and routinely processed for paraffin embedding. Four micrometer thick sections were stained with hematoxylin and eosin. Evaluation included identification of cell types, extent and location of the infiltration, presence of edema, hemorrhage, and necrosis. Tumor grading was based on number of mitotic figures, cell density, and cell anaplasia according to the human 2007 WHO classification of tumors of the central nervous system and current veterinary literature (27–30). Diagnostic yield was defined as the number of biopsy procedures that allowed a histopathologic diagnosis (19, 31). Diagnostic accuracy was defined as the number of biopsy procedures that gave the same diagnosis as necropsy specimen examination. In the latter cases, the brains were removed from the skull immediately after the post mortem procedure.

### Statistical Analyses

Needle placement error data is reported as median and range. The correlation of experience with time in both intra vitam and post mortem procedures was evaluated with the Spearman's rank correlation coefficient, the influence of intra vitam vs. post mortem biopsies with the Kruskal-Wallis one-way ANOVA on ranks. *P* values < 0.05 were considered statistically significant. All analyses were performed using commercial statistical software (NCSS version 12.0.2, Kaysville, UT, USA).

## Results

Needle placement error was tested in six cadavers, three male neutered dogs (one French Bulldog, one American Bulldog, and one cross breed) with a median body weight of 21 kg (range: 15–30), and three male neutered cats (two European shorthair, and one Maine Coon) with a median body weight of 5 kg (range: 4.3–5.2) (Table 1). Median piriform and thalamic target site depth in dogs was 54 mm (range 49.5–64.9 mm) and 49.4 mm (range 44–62.5 mm). Median piriform and thalamic target site depth in cats was 31.6 mm (range 23.9–32.2 mm) and 23.5 mm (range 20.5–25.9 mm). In dogs, the calculated median needle placement error was 1.8 mm (range 0.71–2.84 mm) and 1.53 mm (range 1.45–1.99 mm) for piriform lobe and thalamus target sites, respectively. In cats, the calculated median needle placement error was 0.79 mm (range 0.6–1.91 mm) for the piriform lobe target site and 1.29 mm (range 0.47–2.69 mm) for the thalamic target site.

**Table 1:** Signalment, biopsy characteristics, lesion localization, histopathological diagnosis, and outcome.

**Pat. Nr**.	**Species**	**Breed**	**Age (y)**	**Sex**	**Body weight (kg)**	**IV/PM**	**Lesion size (mm)**	**Biopsy plan depth (mm)**	**Nr. of biopsies**	**Diagnostic biopsy**	**Gross pathology**	**Lesion localization**	**Diagnosis**	**Outcome**
1	Dog	Boston Terrier	10	F	11	PM	19.7	38.2	2	Yes	Yes	Intra-axial, multifocal parietal, temporal, occipital and left thalamus	Anaplastic astrocytoma (WHO Grade III)	
2	Dog	Portuguese Water Dog	5	FS	26	PM	16.2	46.5	3	Yes	No	Intra-axial, bilateral multifocal forebrain and brainstem	Necrotising encephalitis	
3	Dog	Boxer	6	M	35	IV	20.6	49.6	5	Yes	No	Intra- and extra-axial, left temporal lobe and left ventricle	Oligodendroglioma	Discharged
4	Dog	Bolonka Zwetna	5	FS	4	PM	26.2	31	2	Yes	Yes	Intra-axial, right frontal lobe	Anaplastic oligodendroglioma (WHO Grade III)	
5	Dog	French Bulldog	7	FS	10.1	IV	13.5	47.9	5	Yes	No	Intra- and extra-axial, right temporal lobe and right lateral ventricle	Anaplastic oligodendroglioma (WHO Grade III)	Discharged
6	Cat	British Shorthair	17	FS	4.8	IV	13.9	17.4	3	Yes	No	Intra-axial, left temporal lobe	Diffuse astrocytoma (WHO Grade II)	Euthanasia
7	Dog	Mixed breed	8	MN	19	PM	18.2	47.6	3	Yes	Yes	Intra-axial and extra-axial, left cerebellar hemisphere, left lateral and fourth ventricles	Choroid plexus carcinoma	
8	Dog	Mixed breed	10	MN	6.9	PM	16.3	30.2	4	Yes	Yes	Intra-axial, right thalamus and mesencephalon	Anaplastic oligodendroglioma (WHO Grade III)	
9	Cat	Birman	5	FS	3.7	PM	15.3	32.5	4	Yes	Yes	Intra-axial, mesencephalon and metencephalon	Anaplastic oligodendroglioma (WHO Grade III)	
10	Dog	Labrador Retriever	11	MN	29.4	PM	14.8	57.1	4	Yes	Yes	Extra-axial, left lateral ventricle	Choroid plexus carcinoma	
11	Dog	Golden Retriever	6	M	40.1	PM	20.2	28.5	4	Yes	Yes	Intra-axial, left thalamus	Hystiocytic sarcoma	
12	Dog	Shih Tzu	10	FS	8.9	PM	18.8	23.4	3	Yes	Yes	Intra-axial, left temporal lobe	Cystic metastatic carcinoma	
13	Dog	Poodle	11	FS	9.5	PM	13.8	37.2	3	Yes	Yes	Intra-axial, bilateral multifocal forebrain	Vasculitis and meningoencephalitis of unknown origin	
14	Dog	Magyar Viszla	1	M	26.2	IV	21.7	37.2	4	Yes	Yes	Intra- and extra-axial, right thalamus and right lateral ventricle	Meningioma and Meningoangiomatosis	Euthanasia
15	Dog	Collie	10	M	20	IV	20.9	20.1	6	Yes	No	Extra-axial, right rostral cranial fossa, extracranial extension	Transitional meningioma (WHO Grade I)	Euthanasia
16	Dog	Border Terrier	8	F	10.8	PM	12.7	35.9	4	Yes	Yes	Intra-axial, left frontal lobe	Anaplastic oligodendroglioma (WHO Grade III)	
17	Dog	French Bulldog	4	F	10.8	PM	19.5	37.8	4	Yes	Yes	Intra-axial, right frontal lobe	Anaplastic oligodendroglioma (WHO Grade III)	
18	Dog	Bolonka Zwetna	8	MN	7.9	PM	23.6	34.3	6	Yes	No	Intra- and extra-axial, left frontal lobe	Atypical meningioma (WHO Grade II)	
19	Dog	French Bulldog	5	M	15.5	PM	25.9	27.8	4	Yes	Yes	Intra- and extra-axial, left frontal lobe and left lateral ventricle	Oligodendroglioma (WHO Grade III)	
20	Cat	European shorthair	7	FS	6	PM	12.1	28.2	3	Yes	Yes	Extra-axial, left rostral cranial fossa, extracranial extension	Sarcoma	
21	Dog	Bernese Mountain Dog	6	M	55	PM	15	58.2	3	Yes	Yes	Intra-axial, bilateral multifocal forebrain	Granulomatous and necrotizing meningoencephalitis	
22	Dog	Mixed breed	12	MN	40	PM	11	55.4	3	Yes	No	Extra-axial, left lateral ventricle	Choroid plexus-tumor (unclassified grade)	
23	Dog	Boxer	7	FS	26	PM	32.6	47.5	4	Yes	Yes	Intra-axial, right temporal lobe	Oligodendroglioma (WHO Grade II)	
24	Dog	Boxer	1	M	24	PM	22.7	60	3	No	No	Intra-axial, left temporal lobe	Anaplastic oligodendroglioma (WHO Grade III)	
25	Dog	German shepherd	9	FS	32	IV	18.8	20.8	2	Yes	No	Intra-axial, left cerebellar hemisphere	Diffuse astrocytoma (WHO Grade II)	Discharged
26	Dog	Dachshund	6	M	6.5	PM	14	53	4	Yes	Yes	Intra-axial, right thalamus	Granulomatous encephalitis and vasculitis	
27	Cat	European Shorthair	13	FS	4	PM	11.7	46.1	4	Yes	No	Extra-axial, sella	Chromophobe hypophyseal adenoma	
28	Dog	Golden Retriever	8	FS	27	IV	18.4	43.1	3	Yes	No	Intra-axial, right occipital lobe	Unspecific lymphoplasmacytic encephalitis	Discharged

*Pat., patient; Nr., number; F, female; FS, female spayed; M, male; MN, male neutered; y, years; kg, kilogram; IV, intra vitam; PM, post mortem; MRI, magnetic resonance imaging; CT, computed tomography; mm, millimeters; WHO, World Health Organization*.

Frameless image-guided stereotactic brain biopsies were performed in twenty-eight animals. Twenty-one procedures were performed post mortem (eighteen dogs, three cats), and seven biopsy procedures were performed in alive patients (six dogs, one cat). Post mortem biopsies were performed within 8 h of euthanasia. Biopsy procedures in alive animals were performed between three to seven days after initial diagnosis. In all but one animal, the intracranial lesion was diagnosed on MRI, in one dog (Patient number 15) a CT of the head was performed. Demographics and lesion characteristics are summarized in Table 1. Dog breeds included Boxer (3), French Bulldog (3), Bolonka Zwetna (2), Golden Retriever (2), Bernese Mountain Dog (1), Border Terrier (1), Boston Terrier (1), Collie (1), Dachshund (1), German Shepherd (1), Labrador Retriever (1), Magyar Viszla (1), Poodle (1), Portuguese Water Dog (1), Shih Tzu (1), and Cross Breed (3). Three dogs were intact female, eight spayed female, eight intact male, and five neutered male. Cat breeds included European Shorthair (2), Birman (1), and British shorthair (1). All cats were female spayed. The median age was 8 years (range 1–12 years) in dogs and 10 years (range 5–17 years) in cats. The median body weight was 19.5 kg (range 4–55 kg) in dogs and 4.4 kg (range 3.7–4.8 kg) in cats. Canine brain lesions were intra-axial in fifteen cases, extra-axial in three, and both intra- and extra-axial in six. Feline brain lesions were intra-axial and extra-axial in two cases each. The median maximum lesion diameter was 18.8 mm in dogs (range 11–32.6 mm) and 17.25 mm (range 11.7–15.3 mm) in cats. In all animals but two dogs, where MRI was performed post mortem, the lesions showed contrast enhancement on MRI (25/28) and CT (1/1).

The median target site depth in dogs was 38 mm (range 20.1–60 mm) and 30.35 mm in cats (range 17.4–46.1 mm). Biopsies were taken along a single trajectory in all cases. Surgical approach was left rostrotentorial in thirteen cases, right rostrotentorial in twelve cases, and caudotentorial in two cases. The median number of biopsies was four in dogs (range 2–6 biopsies) and four in cats (range 3–4 biopsies).

Gross pathologic specimens were available for comparison in 18/28 animals. Biopsies allowed a histopathologic diagnosis in 27/28 animals (96.4% 95% CI 0.81–0.99). The single failed biopsy procedure was due to insufficient amount of tissue. The biopsy diagnosis was confirmed by pathology specimen histopathology in seventeen of eighteen cases (94.4%, 95% CI 0.72–0.99).

### Procedural Duration

**Figure 5:**
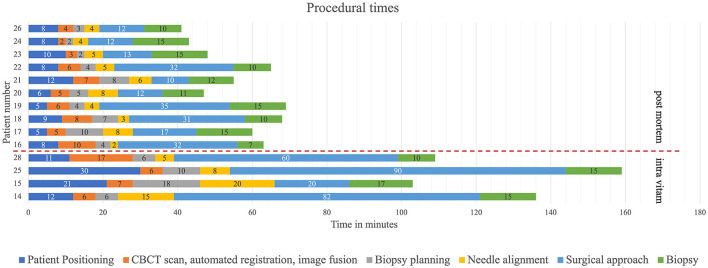
Procedural time intervals with color coding (dark blue = patient positioning, orange = CBCT scan, automated fusion and image fusion, gray = biopsy planning, yellow needle alignment, light blue = surgical approach, green = biopsy) for post mortem (above the interrupted red line) and intra vitam patients (below the interrupted red line).

Time intervals were recorded for four procedures in living animals and ten post mortem biopsy procedures (Figure 5) Median total procedural duration for intra vitam biopsies was 122.5 min (range 103–136 min). Median total procedural duration for post mortem biopsies was 57.5 min (range 41–69 min). The Spearman's rank correlation test showed that with experience there was a significant shortening of the total procedural duration (*p* = 0.03), O-arm acquisition, merge and automated registration (*p* = 0.02), and biopsy planning for the post mortem biopsies (*p* = 0.04). Kruskal-Wallis one-way ANOVA on ranks showed total procedure duration (*p* = 0.005), patient positioning (*p* = 0.009), needle alignment (*p* = 0.03), and surgical approach (*p* = 0.02) for post mortem biopsies to be significantly shorter than for intra vitam biopsies (Figure 5).

### Outcome of Intra Vitam Biopsy Procedures

Seven biopsy procedures were performed in living patients. Two dogs were euthanized within 24 h after the biopsy procedure because of failure to regain spontaneous respiration. Both dogs were comatose and showed signs of increased intracranial pressure on MRI prior to the biopsy procedure. Despite associated high surgical risk, biopsies had been taken at the owner's request. One cat developed decompensation of subclinical hypertrophic cardiomyopathy that did not respond to medical treatment and was euthanized 48 h after biopsy procedure. Three dogs were discharged 1 day after the biopsy, one dog after 6 days (Table 1).

## Discussion

Diagnosis and specific treatment planning for intracranial disease requires tissue sampling and histopathologic examination (6). Inaccurate diagnosis may lead to inappropriate therapy and incorrect prognosis. Likewise, meaningful evaluation of new treatment protocols should ideally be based on histologic diagnosis in order to avoid erroneous conclusions (31). Tissue sampling is not routinely performed in companion animals due to lack of equipment and expertise, technical difficulties associated with the variable skull morphology, size and thick masticatory muscle cover, as well as owner perception of the risks and high costs associated with the procedure. To overcome part of these hurdles and in an attempt to simplify the brain biopsy procedure in dogs and cats, we tested an intraoperative CBCT-based automated registration system for frameless brain biopsies that obviates user-dependent patient registration and transport between the imaging and operating suites, two intermediate steps that have the potential to decrease target accuracy and prolong procedural time.

Frameless stereotaxy patient registration based on anatomical or skin fiducials has been shown to produce comparable results in people but are considered to be highly operator dependent. Modern mobile intraoperative computed tomography systems allow user-independent immediate and automatic patient registration with high accuracy. Navigated surgery using the O-arm in combination with the Stealth Station has been successfully implemented in cranial and spinal interventions in people and recently for orthopedic interventions in equines (24). Cranial interventions are reported for endonasal surgeries of the skull base, hypophysectomy, and electrode placement for deep brain stimulation (32, 33). The reported Euclidean vector error between 1.59 and 2.16 for electrode placement is similar to the median needle placement error obtained in the present study (25). Considering the maximum needle placement error of 2.84 mm we obtained in one dog, diagnostic biopsies can be obtained from lesions with diameters as small as 5.7 mm. Such target accuracies are usually acceptable for brain biopsy samples in veterinary medicine with reported values ranging from 0.9 to 4.3 mm using a variety of frame-based and frameless systems (17, 20, 21, 34). The intraoperative CBCT-based automated registration system for frameless brain biopsies used in the present study can therefore be considered a valid alternative approach.

Apart from target accuracy, diagnostic yield and diagnostic accuracy are the most important criteria to evaluate success of brain biopsies. In the present study, the diagnostic yield was 96.4% and the diagnostic accuracy was 94.4%. These results compare favorably with the limited data available in veterinary medicine (35) where the diagnostic yield varies between 82 to 100% for free-hand and frame-based biopsies in dogs with encephalitis (10, 36), and from 81 to 94.6% in dogs with neoplastic disease and frame-based systems (10, 17, 19). Diagnostic accuracy has been reported to range from 13% using a free-hand biopsy technique (37), to 81% (19) and 91% (10), and therefore we consider the reported system to be adequate. The single non-diagnostic sample consisted of mucinous fluid and the sample was insufficient for diagnosis. This dog was subsequently diagnosed as a grade III oligodendroglioma on necropsy. The failure to deliver adequate amounts of tissue was attributed to the inability to preserve the integrity of the gelatinous tumor matrix despite use of a nylon mesh biopsy bag and a biopsy cassette rather than needle placement error. Similarly, in another dog (patient number three) a diagnosis of oligodendroglioma could be made, but sample quality was insufficient for grading. In the remaining cases diagnostic biopsies could be retrieved even in poorly accessible locations such as deep-seated mid-line, brainstem, and cerebellar sites. Lesion size, depth, location, and number of biopsies did not have an effect on diagnostic yield or diagnostic accuracy.

Still considered the standard of care due to its excellent accuracy, the use of frame-based systems in veterinary patients has disadvantages resulting from the bulky frame that needs to be firmly attached to the skull. Large differences in skull size, morphology, and thickness of the masticatory musculature among different canine breeds and small skull size among felines poses a challenge and there is a need for simpler, less time consuming, and safe alternatives. A recently developed method utilizing 3D printed brain personalized biopsy devices ingenuously addresses variable skull morphology and size but still requires an additional anesthesia for surgical implantation of superficial bone anchors before the actual biopsy procedure (20, 38). The intraoperative imaging capability of the mobile CBCT unit has the advantage that after the initial diagnostic MRI scan, further imaging and biopsy procedures are performed during the same anesthesia. Target planning can be performed as soon as the MRI sequence is loaded onto the workstation and modified at any time during the procedure as needed. The automatic image fusion of the CBCT volume with the MRI sequence worked reliably, and since the patient reference array and reflecting markers on the CBCT unit are recognized by the workstation's infrared camera, no manual patient registration is necessary after volume acquisition. These features eliminate two possible sources of error and potentially reduce procedural time. In the present study median total procedural time in alive patients was 122.5 min and therefore within the range of previously reported duration times of 60–240 min (10, 11, 17). Because time savings associated with frameless stereotaxy have been supported by numerous studies (39, 40), we expected even shorter procedural times. The surgeon's lack of experience and training, both crucial factors to short procedural times, might explain some of the delay (10). Indeed, we noticed a shortening of procedural time with increasing experience for postmortem biopsies from 69 to 41 min. We expect analysis of a larger number of both intra vitam and post mortem procedures with the present system to reveal time savings more clearly. Comparison of brain biopsy procedural times in companion animals is problematic as published data generally do not clearly define start and endpoints (10, 11, 17, 21), or consider time spent on preparatory imaging, which is often done on separate days. For example, if defined as incision-to-end of biopsy retrieval median procedural times for intra vitam and post mortem procedures would be as low as 78.5 and 30 min, respectively, in this study. We opted to report individual time intervals and computed total procedural time by adding individual time intervals to provide a better overview knowing that comparison with previous studies would be difficult at best.

The low case numbers of alive dogs and cats precluded evaluation of experience on intra vitam procedural times. The difference between post mortem compared to intra vitam biopsy procedure times is thought to be due to easier instrument and animal handling.

### Limitations

The present study has several limitations. Target accuracy was based on repeatedly merged images with the biopsy needle in place as described before. The inevitable metal artifacts due to beam hardening on post-operative images were considered acceptable to determine the needle tip location but may have introduced minor inaccuracies. Differences in registration and fusion accuracy when, respectively, merging MRI, CBCT, or CT with intraoperative CBCT cannot be excluded. Additionally, the influence of system inherent, registration, and fusion errors could not be assessed since the details of the image registration and fusion algorithms are not publicly known for commercial proprietary software packages. Needle placement error might therefore ultimately be considered as the summation of these factors in addition to mechanical alignment error. However, the needle placement error determined in the present study is in keeping with previously reported target accuracies in both phantom and clinical studies in people undergoing deep brain stimulation using this system ranging between 0.75 and 1.68 mm (41). Procedural times were not measured in all patients because spatial constraints imposed by operating room size and geometry as well as the need to accommodate anesthetic equipment and additional personnel required adjustments in the disposition of the CBCT, the workstation, as well as of the articulating instrument holder on the surgical table during the first three intra vitam brain biopsy procedures. Besides uneven sample sizes, meaningful statistical comparison between intra vitam and post mortem groups was further hampered by variable skull size, morphology, and lesion location.

## Conclusion

In conclusion, the use of mobile intraoperative CBCT-based registration combined with neuronavigation for frameless brain biopsies in companion animals showed to be an efficient method to sample brain tissue with a good target accuracy, diagnostic yield and diagnostic accuracy independent on skull size and morphology. Studies including a larger number of patients are needed to investigate potential time savings compared to previously published brain biopsy systems.

## Data Availability Statement

The original contributions presented in the study are included in the article/supplementary material, further inquiries can be directed to the corresponding author/s.

## Ethics Statement

Ethical review and approval was not required for the animal study because the study was performed in agreement with the local ethical regulations. Written informed consent was obtained from the owners for the participation of their animals in this study.

## Author Contributions

FM and DS conception and design of the study. FM, AM, AO, and DS-G data acquisition. FF neurosurgical management. FM drafting of the manuscript. All authors contributed to the article and approved the submitted version.

## Conflict of Interest

The authors declare that the research was conducted in the absence of any commercial or financial relationships that could be construed as a potential conflict of interest.

## Publisher's Note

All claims expressed in this article are solely those of the authors and do not necessarily represent those of their affiliated organizations, or those of the publisher, the editors and the reviewers. Any product that may be evaluated in this article, or claim that may be made by its manufacturer, is not guaranteed or endorsed by the publisher.

## References

[1] Rossmeisl JH. New treatment modalities for brain tumors in dogs and cats. Vet Clin North Am Small Anim Pract. (2014) 44:1013–38. 10.1016/j.cvsm.2014.07.003 25441624

[2] Dickinson PJ. Advances in diagnostic and treatment modalities for intracranial tumors. J Vet Intern Med. (2014) 28:1165–85. 10.1111/jvim.12370 24814688 PMC4857954

[3] Cervera V Mai W Vite CH Johnson V Dayrell-Hart B Seiler GS. Comparative magnetic resonance imaging findings between gliomas and presumed cerebrovascular accidents in dogs. Vet Radiol Ultrasound. (2011) 52:33–40. 10.1111/j.1740-8261.2010.01749.x 21322385

[4] Wolff CA Holmes SP Young BD Chen AV Kent M Platt SR. Magnetic resonance imaging for the differentiation of neoplastic, inflammatory, and cerebrovascular brain disease in dogs. J Vet Intern Med. (2012) 26:589–97. 10.1111/j.1939-1676.2012.00899.x 22404482

[5] Diangelo L Cohen-Gadol A Heng HG Miller MA Hague DW Rossmeisl JH. Glioma mimics: magnetic resonance imaging characteristics of granulomas in dogs. Front Vet Sci. (2019) 6:286. 10.3389/fvets.2019.00286 31555671 PMC6722480

[6] Arbit E Galicich JH. Importance of image-guided stereotactic biopsy to confirm diagnosis in an oncological setting. Ann Surg Oncol. (1994) 1:368–72. 10.1007/BF02303807 7850537

[7] Callovini GM. Is it appropriate to redefine the indication for stereotactic brain biopsy in the MRI Era? Correlation with final histological diagnosis in supratentorial gliomas. Minim Invasive Neurosurg. (2008) 51:109–13. 10.1055/s-2008-1058096 18401825

[8] Friedman WA Sceats DJ Jr Nestok BR Ballinger WE Jr. The incidence of unexpected pathological findings in an image-guided biopsy series: a review of 100 consecutive cases. Neurosurgery. (1989) 25:180–4. 10.1227/00006123-198908000-00005 2671781

[9] LeCouteur RA. Current concepts in the diagnosis and treatment of brain tumours in dogs and cats. J Small Anim Pract. (1999) 40:411–6. 10.1111/j.1748-5827.1999.tb03113.x 10516946

[10] Koblik PD LeCouteur RA Higgins RJ Bollen AW Vernau KM Kortz GD. CT-guided brain biopsy using a modified pelorus mark III stereotactic system: experience with 50 dogs. Vet Radiol Ultrasound. (1999) 40:434–40. 10.1111/j.1740-8261.1999.tb00371.x 10528834

[11] Moissonnier P Blot S Devauchelle P Delisle F Beuvon F Boulha L. Stereotactic CT-guided brain biopsy in the dog. J Small Anim Pract. (2002) 43:115–23. 10.1111/j.1748-5827.2002.tb00041.x 11916055

[12] Flegel T Podell M March PA Chakeres DW. Use of a disposable real-time CT stereotactic navigator device for minimally invasive dog brain biopsy through a mini-burr hole. AJNR Am J Neuroradiol. (2002) 23:1160–3. 12169475 PMC8185720

[13] Koblik PD LeCouteur RA Higgins RJ Fick J Kortz GD Sturges BK. Modification and application of a pelorus mark III stereotactic system for CT-guided brain biopsy in 50 dogs. Vet Radiol Ultrasound. (1999) 40:424–33. 10.1111/j.1740-8261.1999.tb00370.x 10528833

[14] Giroux A Jones JC Bøhn JH Duncan RB Waldron DR Inzana KR. A new device for stereotactic CT-guided biopsy of the canine brain: design, construction, and needle placement accuracy. Vet Radiol Ultrasound. (2002) 43:229–36. 10.1111/j.1740-8261.2002.tb00995.x 12088316

[15] Taylor AR Cohen ND Fletcher S Griffin JF Levine JM. Application and machine accuracy of a new frameless computed tomography-guided stereotactic brain biopsy system in dogs. Vet Radiol Ultrasound. (2013) 54:332–42. 10.1111/vru.12025 23551960

[16] Squires AD Gao Y Taylor SF Kent M Tse ZT. A simple and inexpensive stereotactic guidance frame for MRI-guided brain biopsy in canines. J Med Eng. (2014) 2014:139535. 10.1155/2014/139535 27006928 PMC4782635

[17] Rossmeisl JH Andriani RT Cecere TE Lahmers K LeRoith T Zimmerman KL. Frame-based stereotactic biopsy of canine brain masses: technique and clinical results in 26 cases. Front Vet Sci. (2015) 2:20. 10.3389/fvets.2015.00020 26664949 PMC4672202

[18] Packer RA Freeman LJ Miller MA Fauber AE Morrison WB. Evaluation of minimally invasive excisional brain biopsy and intracranial brachytherapy catheter placement in dogs. Am J Vet Res. (2011) 72:109–21. 10.2460/ajvr.72.1.109 21194343

[19] Kani Y Cecere TE Lahmers K LeRoith T Zimmerman KL Isom S. Diagnostic accuracy of stereotactic brain biopsy for intracranial neoplasia in dogs: comparison of biopsy, surgical resection, and necropsy specimens. J Vet Intern Med. (2019) 33:1384–91. 10.1111/jvim.15500 30990928 PMC6524398

[20] Gutmann S Winkler D Müller M Möbius R Fischer JP Böttcher P. Accuracy of a magnetic resonance imaging-based 3D printed stereotactic brain biopsy device in dogs. J Vet Intern Med. (2020) 34:844–51. 10.1111/jvim.15739 32091636 PMC7096628

[21] Troxel MT Vite CH. CT-guided stereotactic brain biopsy using the Kopf stereotactic system. Vet Radiol Ultrasound. (2008) 49:438–43. 10.1111/j.1740-8261.2008.00403.x 18833950

[22] Dorward NL Paleologos TS Alberti O Thomas DG. The advantages of frameless stereotactic biopsy over frame-based biopsy. Br J Neurosurg. (2002) 16:110–8. 10.1080/02688690220131705 12046728

[23] Maciunas RJ Galloway Jr RL Latimer JW. The application accuracy of stereotactic frames. Neurosurgery. (1994) 35:682–94; discussion 94–5. 10.1097/00006123-199410000-00015 7808612

[24] de Preux M Klopfenstein Bregger MD Brunisholz HP Van der Vekens E Schweizer-Gorgas D Koch C. Clinical use of computer-assisted orthopedic surgery in horses. Vet Surg. (2020) 49:1075–87. 10.1111/vsu.13486 32677115

[25] Sharma M Deogaonkar M. Accuracy and safety of targeting using intraoperative “O-arm” during placement of deep brain stimulation electrodes without electrophysiological recordings. J Clin Neurosci. (2016) 27:80–6. 10.1016/j.jocn.2015.06.036 26778050

[26] Carl B Bopp M Sass B Nimsky C. Intraoperative computed tomography as reliable navigation registration device in 200 cranial procedures. Acta Neurochir. (2018) 160:1681–9. 10.1007/s00701-018-3641-6 30051160

[27] Louis DN Ohgaki H Wiestler OD Cavenee WK Burger PC Jouvet A. The 2007 WHO classification of tumours of the central nervous system. Acta Neuropathol. (2007) 114:97–109. 10.1007/s00401-007-0243-4 17618441 PMC1929165

[28] Higgins RJ Bollen AW Dickinson PJ Sisó-Llonch S. Tumors of the nervous system. Tumors Dom Ani. (2016) 834–91. 10.1002/9781119181200.ch1925855820

[29] Merickel JL Pluhar GE Rendahl A O'Sullivan MG. Prognostic histopathologic features of canine glial tumors. Vet Pathol. (2021) 58:945–51. 10.1177/03009858211025795 34219560 PMC10923237

[30] Koehler JW Miller AD Miller CR Porter B Aldape K Beck J. A revised diagnostic classification of canine glioma: towards validation of the canine glioma patient as a naturally occurring preclinical model for human glioma. J Neuropathol Exp Neurol. (2018) 77:1039–54. 10.1093/jnen/nly085 30239918 PMC6181180

[31] Jackson RJ Fuller GN Abi-Said D Lang FF Gokaslan ZL Shi WM. Limitations of stereotactic biopsy in the initial management of gliomas. Neuro Oncol. (2001) 3:193–200. 10.1093/neuonc/3.3.193 11465400 PMC1920616

[32] Banat M Wach J Salemdawod A Bahna M Scorzin J Vatter H. The role of intraoperative image guidance systems (Three-Dimensional C-arm versus O-arm) in spinal surgery: results of a single-center study. World Neurosurg. (2021) 146:e817–21. 10.1016/j.wneu.2020.11.013 33181376

[33] Krahulik D Nevrly M Otruba P Bardon J Hrabalek L Pohlodek D. O-arm navigated frameless and fiducial-less deep brain stimulation. Brain Sci. (2020) 10:683. 10.3390/brainsci10100683 32992610 PMC7600133

[34] Chen AV Wininger FA Frey S Comeau RM Bagley RS Tucker RL. Description and validation of a magnetic resonance imaging-guided stereotactic brain biopsy device in the dog. Vet Radiol Ultrasound. (2012) 53:150–6. 10.1111/j.1740-8261.2011.01889.x 22122485

[35] Dhawan S He Y Bartek Jr J Alattar AA Chen CC. Comparison of frame-based versus frameless intracranial stereotactic biopsy: systematic review and meta-analysis. World Neurosurg. (2019) 127:607–16.e4. 10.1016/j.wneu.2019.04.016 30974279

[36] Flegel T Oevermann A Oechtering G Matiasek K. Diagnostic yield and adverse effects of MRI-guided free-hand brain biopsies through a mini-burr hole in dogs with encephalitis. J Vet Intern Med. (2012) 26:969–76. 10.1111/j.1939-1676.2012.00961.x 22708694

[37] Harari J Moore M Leathers C Roberts G Gavin P. Computed tomographic-guided, free-hand needle biopsy of brain tumors in dogs. Prog Vet Neurol. (1993) 4:41–4.

[38] Müller M Winkler D Möbius R Sauerstein T Scholz S Gutmann S. A concept for a 3D-printed patient-specific stereotaxy platform for brain biopsy -a canine cadaver study. Res Vet Sci. (2019) 124:79–84. 10.1016/j.rvsc.2019.02.007 30856434

[39] Grunert P Espinosa J Busert C Günthner M Filippi R Farag S. Stereotactic biopsies guided by an optical navigation system: technique and clinical experience. Minim Invasive Neurosurg. (2002) 45:11–5. 10.1055/s-2002-23576 11932818

[40] Dammers R Haitsma IK Schouten JW Kros JM Avezaat CJ Vincent AJ. Safety and efficacy of frameless and frame-based intracranial biopsy techniques. Acta Neurochir. (2008) 150:23–9. 10.1007/s00701-007-1473-x 18172567

[41] Katisko JP Kauppinen MT Koivukangas JP Heikkinen ER. Stereotactic operations using the o-arm. Stereotact Funct Neurosurg. (2012) 90:401–9. 10.1159/000341699 23075522

